# SILAC-Based Proteomic Profiling of the Human MDA-MB-231 Metastatic Breast Cancer Cell Line in Response to the Two Antitumoral Lactoferrin Isoforms: The Secreted Lactoferrin and the Intracellular Delta-Lactoferrin

**DOI:** 10.1371/journal.pone.0104563

**Published:** 2014-08-12

**Authors:** Esthelle Hoedt, Karima Chaoui, Isabelle Huvent, Christophe Mariller, Bernard Monsarrat, Odile Burlet-Schiltz, Annick Pierce

**Affiliations:** 1 UGSF, UMR 8576 CNRS, USTL, IFR 147, Villeneuve d'Ascq, France; 2 CNRS, IPBS (Institut de Pharmacologie et de Biologie Structurale), Toulouse, France; 3 Université de Toulouse, UPS, IPBS, Toulouse, France; Virginia Commonwealth University, United States of America

## Abstract

**Background:**

Lactoferrins exhibit antitumoral activities either as a secretory lactoferrin or an intracellular delta-lactoferrin isoform. These activities involve processes such as regulation of the cell cycle and apoptosis. While lactoferrin has been shown to exert its function by activating different transduction pathways, delta-lactoferrin has been proven to act as a transcription factor. Like many tumor suppressors, these two proteins are under-expressed in several types of cancer, particularly in breast cancer.

**Methodology/Principal Findings:**

In order to compare the differential effects of the re-introduction of lactoferrin isoforms in breast cancer cells we chose the cancerous mammary gland MDA-MB-231 cell line as a model. We produced a cell line stably expressing delta-lactoferrin. We also treated these cells with fresh purified human breast lactoferrin. We performed two quantitative proteomic studies in parallel using SILAC coupled to mass spectrometry in order to compare the effects of different doses of the two lactoferrin isoforms. The proteome of untreated, delta-lactoferrin expressing and human lactoferrin treated MDA-MB-231 cells were compared. Overall, around 5300 proteins were identified and quantified using the in-house developed MFPaQ software. Among these, expression was increased by 1.5-fold or more for around 300 proteins in delta-lactoferrin expressing cells and 190 proteins in lactoferrin treated cells. At the same time, about 200 and 40 proteins were found to be downregulated (0-0.7-fold) in response to delta-lactoferrin and lactoferrin, respectively.

**Conclusions/Significance:**

Re-introduction of delta-lactoferrin and lactoferrin expression in MDA-MB-231 mainly leads to modifications of protein profiles involved in processes such as proliferation, apoptosis, oxidative stress, the ubiquitin pathway, translation and mRNA quality control. Moreover, this study identified new target genes of delta-lactoferrin transcriptional activity such as *SelH*, *GTF2F2* and *UBE2E1*.

## Introduction

Over the last decade, it has become clear that lactoferrin isoforms have a role as anti-tumoral agents and behave as tumor suppressors. Lactoferrins exist as different variants due to gene polymorphisms, post-transcriptional and post-translational modifications. The two main isoforms are secreted Lf (Lf) [1] and its nucleocytoplasmic counterpart, delta-lactoferrin (ΔLf) [2], [3]. Their expression is downregulated or silenced in cancer cells [2], [4], [5]. In some cancers, significantly lower levels of Lf and/or ΔLf correlated with more advanced disease and an unfavourable prognosis [4], [6]. This downregulation is mainly due to genetic and epigenetic modifications which have been found on the *Lf* gene in some forms of cancer [7], [8].

Lf and ΔLf mRNAs are derived from the transcription of the *Lf* gene at alternative promoters [3]. Their translation leads to Lf, an 80 kDa iron-binding protein widely distributed in biological fluids and also expressed by immune cells [9], [10] and to ΔLf, a 73 kDa intracellular protein in which the leader sequence and the first 25 amino acid residues of Lf are absent [11]. Both isoforms possess NLS motifs [12], [13] and the use of a GFP-ΔLf fusion protein clearly demonstrated that ΔLf targets the nucleus [3], [13], [14], [15] whereas the nuclear targeting of Lf is still controversial [3], [14], [16], [17], [18], [19]. Thus, uptake and nuclear targeting of Lf depend on its target cells and on the presence of specific mammalian receptors (LfRs) at their surfaces such as LRP, CD14, nucleolin and intelectin [16], [17]. The two basic regions of Lf, described as putative DNA binding domains [20], are present on ΔLf and are good candidates for their interaction with DNA sequences. As a secreted protein, Lf is *N*-glycosylated [21] whereas ΔLf is *O*-GlcNAcylated [15]. The role of the Lf glycan moiety seems to be restricted to a decrease in the immunogenicity of the protein and its protection from proteolysis [22]. On the other hand, *O*-GlcNAcylation positively regulates ΔLf stability and negatively regulates its transcriptional activity [15].

Lf limits cell proliferation and migration. Oral administration of Lf reduces tumor growth and the number of metastases in numerous animal models of chemically induced carcinogenesis and transplanted tumors [23], [24], [25]. Recently, it was shown that bovine Lf (bLf) inhibits colorectal cancer in animal models and that human Lf (hLf) reduced the risk of colon cancer [26]. Camel milk Lf reduces the proliferation of colorectal cancer cells and also exerts antioxidant and DNA damage inhibitory activities [27]. Lf acts in many ways to control the G1/S transition in malignant cells such as the breast cancer MDA-MB-231 cell lines [28]. Blocking the transition from G1 to S mainly targets the MAPK pathway with decreased phosphorylation of AKT, hypophosphorylation of Rb, overexpression of p27 and cyclin E and under-expression of cyclin D [29], [30]. Recently, it was shown that modulation of Lf levels in nasopharyngeal carcinoma cells affects their proliferation and invasiveness phenotypes by interfering with the MAPK signaling pathway *via* a downregulation of both the levels of PDK1 and keratin K18-mediated AKT activation [31]. Activation of the NF-kB pathway followed by the overexpression of p53, p21 and mdm2 has also been described [32]. In HeLa cells, Lf induces growth arrest and nuclear accumulation of Smad-2 *via* the TGFβ/Smad-2 pathway [33].

Lf also functions as a biological mediator of apoptosis [34]. *In vivo* studies have shown that oral administration of bLf inhibits tumorigenesis and enhances apoptosis by inducing the expression of the death receptor Fas and pro-apoptotic proteins Bax and Bid, activation of caspases 8 and 3 and induction of DNA fragmentation [35]. *In vitro* studies have shown that Lf promotes apoptosis in the human leukemia Jurkat T-cell line through efficient cleavage of caspases 9 and 3 and PARP *via* the activation of the JNK signaling pathway [36]. Moreover when high doses of hLf are used, Lf exploits the control mechanism of E2F1-regulated target genes and Bcl-2 family gene networks to trigger the apoptotic process [37]. On the other hand, studies on neuronal PC12 cells showed that hLf can promote or inhibit apoptosis depending on the applied dose [38]. Recently, adenoviruses encoding hLf were used to explore tumor growth suppression effects. Injection of these adenoviruses directly into tumors induced apoptosis [39]. Adenoviruses were also used on cervical cancer cells *in vitro* and *in vivo* in which a strong tumor growth inhibition caused by cell cycle inhibition at the G2/M phase, an elevated expression of Fas and a decreased ratio of anti- to pro-apoptotic molecules Bcl-2/Bax were observed [40].

ΔLf also exhibits antitumoral activities. We already showed that overexpression of ΔLf leads to cell cycle arrest at the G1/S transition [41] and apoptosis [42]. Whereas Lf mainly acts exogenously on tumor cell growth by modulating different transduction pathways [28]–[33], [35], [36], ΔLf exerts its anti-proliferative and pro-apoptotic activities *via* its role as a transcription factor. Lf isoforms are known to interact with DNA sequences *in vitro* for Lf [43], [44], [45], [46] and *in vivo* for ΔLf [13], [42], [47]. Thus, while it is clear that ΔLf acts as a transcription factor *via* a functional ΔLfRE it is less clear whether Lf possesses the same activity *in vivo*. Lf has been found to affect *IL-1β* [44], *endothelin-1* [45] and *ICAM-1* [46] gene expression.

In our group, we demonstrated that ΔLf is capable of activating the expression of Skp1 [13], a protein belonging to Skp1-Cul1-F-box protein (SCF) complex, one of the most well characterized types of ubiquitin ligase (E3), DcpS [47], a pyrophosphatase responsible for mRNA decapping and Bax [42], a pro-apoptotic component. Recently, a genome-wide pathway analysis which compared the different signaling pathways triggered by Lf and ΔLf in HEK 293 cells treated with Lf or expressing ΔLf has generated a considerable body of information on the molecular features of the re-introduction of Lf isoforms in cancerous cells [48]. Data showed significant up and down-regulation respectively of 74 and 125 genes in Lf-treated cells and 327 and 256 genes in ΔLf-expressing cells. Among them, essential genes and signaling networks responsible for cell survival and apoptosis were affected. Moreover, they showed that ΔLf may directly act on RNA processing of HBB, TRA2B and ATP5C1 transcripts favoring their maturation of pre- to mature mRNAs.

Although it is now clear that both Lf isoforms have anti-proliferative and pro-apoptotic activities, the mechanism(s) by which they act are still controversial. For this reason we undertook a large scale proteomic study to identify proteins that are regulated directly or indirectly by Lf isoforms. Changes in the protein expression pattern were investigated by stable isotope labeling by amino acids in cell culture (SILAC) [49], [50], [51]. SILAC depends on metabolic labeling that occurs at the earliest moment in the sample handling process, thereby minimizing errors in quantitation. Therefore, SILAC is the method of choice to describe global protein abundance dynamics when using cell culture systems. It is an elegant way to evaluate the effects of a treatment on a large number of proteins in a single experiment and provides an efficient means of accurate protein quantitation. The proteins were identified by Liquid Chromatography–tandem Mass Spectrometry (LC-MS/MS) and Western blotting and qRT-PCR analyses were performed to confirm corresponding changes in transcript expression. Here we used a triple SILAC in order to compare the differential effects of the re-introduction of Lf or ΔLf versus untreated cells using the cancerous mammary gland MDA-MB-231 cell line as a model. We performed two quantitative proteomic studies in parallel in order to compare the effects of different doses of the two Lf isoforms. Our results showed that re-introduction of Lf or ΔLf expression in MDA-MB-231 cells mainly leads to modifications of protein profiles involved in processes such as proliferation, apoptosis, oxidative stress, ubiquitin pathway, translation and mRNA quality control. Moreover, our study pointed out new target genes of ΔLf transcriptional activity such as the *Selenoprotein H*, the *General Transcription Factor IIF 2* and the *Ubiquitin conjugating enzyme E1* genes.

## Experimental Section

### Establishment of a stable inducible cell line for ΔLf

The stable expression of ΔLf was realized using the tetracyclin inducible Tet-on system (Clontech, Mountain View, CA). The human breast cancer MDA-MB-231 (ATCC HTB-26) cell line was grown in DMEM (Dubelcco's modified Eagle's medium, Thermo Fisher Scientific, Waltham, MA) containing 10% (v/v) FCS, 2 mM L-glutamine and 1% (w/v) penicillin/streptomycin and cultured at 37°C in a humidified atmosphere with 5% CO_2_. MDA-MB-231 cells were further stably transfected with the pTRE responder construct containing the Δ*Lf* cDNA, as described in [41] using the Dreamfect reagent (OZ Biosciences, France) according to the manufacturer's instructions. Isolated clones were expanded to obtain cells named MDA-MB-231 dox-. Expression of ΔLf was followed as described [5]. Clones used for the study did not produce any detectable ΔLf without induction. Stable inducible ΔLf-expressing HEK293 (ATCC CRL-1573), HeLa (ATCC CCL-2) and MCF7 (ATCC HTB 22) cell lines were already available in our laboratory.

### hLf purification

hLf was purified from human milk provided by the milk bank of Jeanne de Flandres Hospital, Lille, France, as described in [52]. Contaminating LPS was removed from hLf using Detoxi-gel (Thermo Fisher Scientific, Bremen, Germany) and then assayed using the *Limulus* amoebocyte lysate assay (QCL1000; BioWhittaker, Walkersville, MD). LPS contamination was under 0.09 E.U/mg hLf.

### SILAC labeling

MDA-MB-231 dox- cells were maintained in stable isotope-labeled DMEM 89985 deficient in lysine and arginine and supplemented with 10% (v/v) dialyzed fetal bovine serum (FBS) (Gibco-Invitrogen), 2 mM L-glutamine, 1% (w/v) penicillin/streptomycin and proline 1% (v/v) to avoid the conversion of labeled arginine to proline (all from Gibco-Invitrogen and Thermo Fisher Scientific) and filtered (0.45 µm, d.i). Cells were grown as usual but in the presence of arginine (L-Arg ^12^C_6_-^14^N_4_) and lysine (L-Lys ^12^C_6_-^14^N_2_) for the light condition, arginine (L-Arg ^13^C_6_-^14^N_4_) and lysine (L-Lys ^13^C_6_-^14^N_2_) for the medium condition or arginine (L-Arg ^13^C_6_-^15^N_4_) and lysine (L-Lys ^13^C_6_-^15^N_2_) for the heavy condition during 8 cellular divisions prior to stimulation. Arginine and lysine concentrations were 84 µg/µL for L-Arg and 146 µg/µL for L-Lys (Cambridge Isotope Laboratories, Inc., Andover, MA). Total metabolic incorporation was checked with mass spectrometry.

### Treatment of cells

Six million MDA-MB-231 dox- cells were seeded into 75 cm^2^ dishes and grown for 24 h. One cell-dish grown in medium SILAC medium was stimulated with 2 µg/mL of doxycycline (Clontech) or transiently transfected with ΔLf-expressing pcDNA vector (1 µg plasmid/10^6^ cells) as described [15]. Another cell-dish grown in heavy SILAC medium was treated with hLf at a concentration of either 50 or 500 µg/mL. The third cell-dish grown in light SILAC medium corresponds to the unstimulated or untreated cell population. For the high dose treatment, in order to eliminate from the analyses all protein expression variations due to the transfection itself, untreated and 500 µg/mL hLf treated cells were also submitted to the transfection agent under the same conditions as the ΔLf transfected cells.

### Subcellular fractionation

At 24 h post-induction, cells grown in each of the three conditions, light, medium, heavy were harvested and mixed at a 1/1/1 ratio. For each SILAC experiment, 5 10^6^ cells were used per condition and rinsed with PBS. Subcellular fractionation was carried out as described [53] with some modifications. Cells were suspended in 500 µl of buffer A [Tris/HCl 50 mM pH 7.5; NaCl 137.5 mM; Triton X100 0.5% (w/v); Glycerol 10% (v/v)] with protease inhibitors (Complete Protease Inhibitor Cocktail, Roche) and incubated for 15 min on ice. The sample was then centrifuged at 13,000 rpm (Heraeus, Biofuge 15R1, HFA 22.2 rotor, 12,000 g) for 15 min at 4 °C. The supernatant corresponds to the cytosolic fraction. The nuclear pellet was washed twice by adding 500 µl of buffer A again (same conditions of centrifugation). Two hundred microliters of buffer B [Tris/HCl 50 mM pH 7.5; NaCl 300 mM; Triton X100 0.5% (w/v); Glycerol 10% (v/v)] were used to resuspend the pellet. The nuclear pellet was sonicated (8×4 sec × force 4 with the *Branson 150* Sonifier) on ice. The supernatant, centrifuged at 13,000 rpm for 15 min at 4 °C, corresponds to the nuclear fraction.

### Analysis of samples by SDS-PAGE

Reduction and alkylation of cysteine residues were performed by diluting 100 µg of each sample in Laemmli buffer for 5 min at 95 °C followed by a treatment with 90 mM iodoacetamide for 30 min at room temperature in the dark. The samples were separated on 4–12% Bis-Tris gels (Invitrogen). Proteins were visualized by Coomassie Blue staining. Each lane was cut into 20 homogenous slices that were washed in 100 mM ammonium bicarbonate for 15 min at 37 °C followed by a second wash in 100 mM ammonium bicarbonate, acetonitrile (1∶1) for 15 min at 37 °C. Proteins were digested by incubating each gel slice with 1 µg of modified sequencing grade trypsin (Promega, Madison, WI) in 50 mM ammonium bicarbonate overnight at 37 °C. The resulting peptides were extracted from the gel by three steps: incubation in 50 mM ammonium bicarbonate for 15 min at 37 °C and two incubations in 10% formic acid, acetonitrile (1∶1) for 15 min at 37 °C. The three collected extractions were pooled with the initial digestion supernatant, dried in a SpeedVac, and resuspended with 14 µl of 2% acetonitrile, 0.05% trifluoroacetic acid.

### LC-MS/MS analysis

Each fraction was analyzed by nanoLC-MS/MS using an Ultimate3000 system (Dionex, Amsterdam, The Netherlands) coupled to an LTQ-Orbitrap Velos ETD mass spectrometer (Thermo Fisher Scientific, Bremen, Germany). Five microliters of the sample were loaded on a C18 precolumn (300 µm ID×5 mm, Dionex) at 20 µL/min in 5% acetonitrile, 0.05% trifluoroacetic acid. After 5 min desalting, the precolumn was switched online with in-house packed column (15 cm reversed-phase capillary emitter column: inner diameter 75 mm, ReproSil-Pur C18-AQ, 3 µm resin), equilibrated in 95% solvent A (5% acetonitrile, 0.2% formic acid) and 5% solvent B (80% acetonitrile, 0.2% formic acid).

Peptides were eluted using a 0 to 50% gradient of solvent B during 105 min at a flow rate of 300 nL/min. The LTQ-Orbitrap was operated in data dependent acquisition mode with the XCalibur software. Survey scan MS were acquired in the Orbitrap in the 350–2000 m/z range with the resolution set to a value of 60,000. The twenty most intense ions per survey scan were selected for collision-induced dissociation (CID) fragmentation and the resulting fragments were analyzed in the linear trap (LTQ). Dynamic exclusion was employed within 60 seconds to prevent repetitive selection of the same peptide.

### Database search and quantitative data analysis

Mascot (version 2.3.01) was used to automatically extract peak lists from raw files. The following parameters were set for creation of the peak lists: parent ions in the mass range 300–4500, no grouping of MS/MS scans. MS/MS data were searched against human sequences in the public database UniProt which consists of Swiss-Prot and TrEMBL. Carbamidomethylation of cysteine residues was set as a fixed modification and oxidation of methionine residues, protein amino terminal acetylation and ^13^C_6_ and/or ^15^N_4_ label on lysine and arginine were set as variable modifications. Specificity of trypsin digestion was set for cleavage after Lys or Arg except before Pro, and two missed trypsin cleavage sites were allowed. The mass tolerances in MS and MS/MS were set to 5 ppm and 0.8 Da, respectively, and the instrument setting was specified as ElectroSpray Ionization (ESI)-Trap. Mascot results were parsed with the in-house developed software Mascot File Parsing and Quantification (MFPaQ) version 4.0 [54], and protein hits were automatically validated if they satisfied one of the following criteria: identification with at least one top ranking peptide with a Mascot score of more than 50 (*p* value <0.001) or at least two top ranking peptides each with a Mascot score of more than 33 (*p* value <0.05).

### Bioinformatics resource

All identified proteins were converted into gene names with the database for annotation, visualization and integrated discovery (DAVID) bioinformatics resource [55], [56]. The PANTHER (protein annotation through evolutionary relationship) classification system [57] was also used in our large-scale proteomics experiments. Up- and downregulated proteins were classified into families and subfamilies of shared functions, which were then categorized by molecular function and biological process ontology terms.

### Western blotting and immunodetection

Cell extracts were prepared from frozen pellets of MDA-MB-231 dox- cells, MDA-MB-231 dox- induced with doxycycline (corresponding to MDA-MB-231-ΔLf cells, low dose), MDA-MB-231 dox- transfected with pcDNA-ΔLf (corresponding to MDA-MB-231-ΔLf cells, high dose), MDA-MB-231 dox- treated with 50 µg/mL hLf (corresponding to MDA-MB-231-hLf cells, low dose), and MDA-MB-231 dox- treated with 500 µg/mL hLf (corresponding to MDA-MB-231-hLf cells, high dose). Subcellular fractionation was performed and protein concentrations were determined using the Bradford assay (Thermo Fisher Scientific). Samples were mixed with 4X Laemmli sample buffer [250 mM Tris/HCl (pH 6.8) containing 20% (v/v) β-mercaptoethanol, 6% (w/w) SDS, 40% (v/v) and 0.04% (w/w) bromophenol blue] and boiled for 5 min. A total of 30 µg of protein of each sample was submitted to SDS-PAGE and analyzed by Western blotting. Blots were subsequently probed with primary antibodies for 2 h at room temperature and secondary antibodies conjugated to horseradish peroxidase (GE Healthcare Life Sciences; Uppsala, Sweden) at 1∶10000 for 1 h, before being detected by chemiluminescence (ECL, GE Healthcare Life Sciences). Monoclonal murine antibody against histone H2B (1∶2000) was purchased from Abcam (Cambridge, UK), polyclonal rabbit anti-glyceraldehyde-3-phosphate dehydrogenase (GAPDH) antibody (1∶1000) from Santa Cruz Biotechnologies Inc. (Dallas, TX). Polyclonal goat antibodies against aldehyde dehydrogenase 18 family member A1 (ALDH18A1), 40S ribosomal protein S9 (RPS9), selenoprotein H (SELH), cathepsin Z (CTSZ) and gamma glutamy hydrolase (GGH) were purchased from Santa Cruz Biotechnologies Inc. Monoclonal rabbit antibody to calmodulin, polyclonal rabbit antibodies to heparanase (HPSE), RNA polymerase-associated protein homolog (RTF1), cytoskeleton-associated protein 4 (CKAP4), eukaryotic translation initiation factor 3 subunit E (eIF3E), acidic leucine-rich nuclear phosphoprotein 32 family member B (ANP32B), ubiquitin-conjugating enzyme E2 E1 (UBE2E1) and general transcription factor IIF subunit 2 (GTF2F2) were purchased from Abcam. Monoclonal mouse zinc finger Ran-binding domain-containing protein 2 (ZNF265) antibody was purchased from Abnova Biotechnologies (Taipei, Taiwan). For SILAC validations, all the antibodies were used according to the manufacturer's instructions. The densitometric analysis was performed using the Quantity One v4.1 software (Bio-Rad, Hercules, CA) or *ImageJ* and statistical analyses were performed with PRISM 5 software (Graphpad, USA).

### DNA and RNA isolation

Genomic DNA was extracted from HEK 293 cells as previously described [13], and purified using the Wizard Genomic DNA Purification kit (Promega, Madison, WI), with the yield assessed by spectrophotometry. All plasmids were purified using the EndoFree Plasmid Kit (Qiagen, Germantown, MD). Total RNA from each condition (MDA-MB-231 dox-; MDA-MB-231-ΔLf, low dose; MDA-MB-231-hLf, low dose; MDA-MB-231-ΔLf, high dose; MDA-MB-231-hLf, high dose) was isolated from cells using a NucleoSpin RNA II kit, according to the instructions of the manufacturer (Macherey-Nagel, Düren, Germany). The purity and integrity of each extract were checked using the nanodrop ND-1000 spectrophotometer (Labtech International, Uckfield, UK) and the Bioanalyzer 2100 (Agilent Technologies, Santa Clara, CA). Reverse transcription was performed from 2 µg of total RNA with an oligo-dT primer and M-MLV reverse transcriptase (Promega).

### Real time PCR

Real-time PCR (qRT-PCR) were performed as described [5] using an Mx3005 thermal cycler system and Brilliant SYBER Green QPCR Master Mix (Stratagene, Agilent Technologies). DNA primer pairs and conditions used to amplify mRNA are compiled in Table S1. The primer pairs were purchased from Eurogentec (Seraing, Belgium). TaqMan qRT-PCR was performed as described [5]. The ΔLf and Lf probes were 5′-FAM-labeled, the normalizing HPRT gene probe was 5′-VIC-labeled (Applied Biosystems, Life Technologies) and the 3′ non-fluorescent quencher (NFQ) (Applied Biosystems) was used for each probe. Relative quantities of targeted mRNA were calculated as described [58] and expressed normalized to hypoxanthine-guanine phosphoribosyltransferase (HPRT). Negative control reactions were performed using sterile water instead of cDNA template. Contaminations of genomic DNA were excluded by performing 35 cycles of amplification without retrotranscription. All qRT-PCR runs were performed in triplicate from three independent assays.

### Reporter gene assays

Reporter gene assays were routinely performed in our laboratory using pcDNA-ΔLf or pcDNA-hLf constructs or a null vector and HEK 293 cells [15], [41], [42]. The reporter pGL3-SelH-Luc vector was obtained as in [13] except that the 167 bp SelH promoter fragment was amplified with the primer pair listed in Table S1, cloned into the pGL3-promoter-Luc vector (Promega) and sequenced before use. HEK 293 cells were transfected (250 ng of DNA for 2×10^5^ cells, 50 ng of reporter vector and 200 ng of ΔLf, hLf expression vector or null vector) using DreamFect (OZ Biosciences, Marseille, France). Cell lysates were assayed using a luciferase assay kit (Promega) in a Tristar multimode microplate reader LB 941 (Berthold Technologies, Bad Wildbab, Germany). Relative luciferase activities were normalized to basal luciferase expression as described [13] and expressed as fold increase to the relative luciferase activity of ΔLf or hLf. Basal luciferase expression was assayed using a null vector and was determined for each vector. Each experiment represents at least three sets of independent triplicates.

### ChIP assays

ChIP assays were routinely performed using a pCMV-3XFLAG-ΔLf and pcDNA-hLf or a null vector and HEK293 cells which were transfected (1 µg of DNA for 1×10^6^ cells) using DreamFect (OZ Biosciences) [13], [15], [41], [42]. The cells were lysed and sonicated using a BIORUPTOR to generate the chromatin preparation, and ChIP assays were performed using The MAGnify Chromatin Immunoprecipitation System kit (Invitrogen) according to the manufacturer's instructions. Chromatin was sonicated to an average size of 400 bp. A small fraction of the sonicated chromatin was put aside before the immunoprecipitation with antibodies, and constituted input DNA. ChIP complexes (2 10^6^ cells) were immunoprecipitated with anti-hLf (Sigma, St Louis, MT), anti-M2 (raised against the 3XFLAG present on 3XFLAG-ΔLf construction, Sigma), or anti-Rabbit IgG (GE Healthcare Life Sciences). The genomic DNA was purified and the recruitment of hLf and ΔLf proteins was measured by real-time qPCR, using specific primer pairs listed in Table S1. The results were normalized with the levels of ΔLfRE present in the samples (input). Data are expressed as fold enrichment related to null-transfected cells, and are the mean ±SD of triplicates from three independent assays. Amplification of the albumin promoter region was used as a negative control (data not shown) [15].

### Cell invasion assay

The ability of MDA-MB-231 cells to pass through Matrigel-coated filters was measured by the Boyden chamber invasion assay as described [45]. The assay was conducted using a 24-well transwell unit (8 µm of pore size) with polyvinylpyrrolidone-free polycarbonate filters coated with 50 µg Matrigel to form a matrix barrier and placed in transwell chambers according to the manufacturer's instructions (BD Biosciences, Bedford, MA, USA). Cells were starved during 24 h and a suspension of 1×105 cells in basal medium containing 0.1% bovine serum albumin (BSA) was added to the upper compartment and incubated with 50 or 500 µg/mL of hLf or vehicle for 24 h at 37 °C. The lower compartment was filled with 400 µL basal medium containing 10% FBS as chemoattractant. After incubation, the cells in the upper surface of the membrane were carefully removed with a cotton swab and cells that invaded across the Matrigel to the lower surface of the membrane were fixed with paraformaldehyde and stained with 1 µg/mL of DAPI. The number of the cells that had migrated was counted using a fluorescence microscope (Zeiss Axioplan 2 imaging system, Carl-Zeiss S.A.S., Le Pecq, France) and the results were expressed as percentages of control and are the mean ±SD of triplicates from three independent assays.

## Results and Discussion

### Large scale proteome analyses

MDA-MB-231, a highly invasive breast cancer cell line known to internalize secreted hLf through interaction with surface nucleolin and to deliver a small amount of it to the nucleus [17], was chosen as a model system to investigate the effect of Lf isoforms, known to share anticancer activities. Since this cancerous cell-line produces very low amounts of Lf isoform transcripts [4], [5], we established a stable and inducible MDA-MB-231 cell line expressing ΔLf under doxycycline induction. These cells were either induced by doxycycline to express ΔLf or treated with two different concentrations of hLf. In order to obtain higher expression levels of ΔLf, these cells were also transfected with a ΔLf expression vector. The low concentration of hLf (50 µg/mL, 0.625 µM) or ΔLf (induction with doxycycline) was used to provoke cell cycle arrest at the G1/S transition [28], [41] and the high concentration of hLf (500 µg/mL, 6.25 µM) or ΔLf (transient transfection with a pcDNA-ΔLf vector) was used to trigger apoptosis [36], [42]. The amount of ΔLf expression vector was adjusted to maintain ΔLf amounts similar to those found in normal NBEC cells [4], [5].

**Figure 1:**
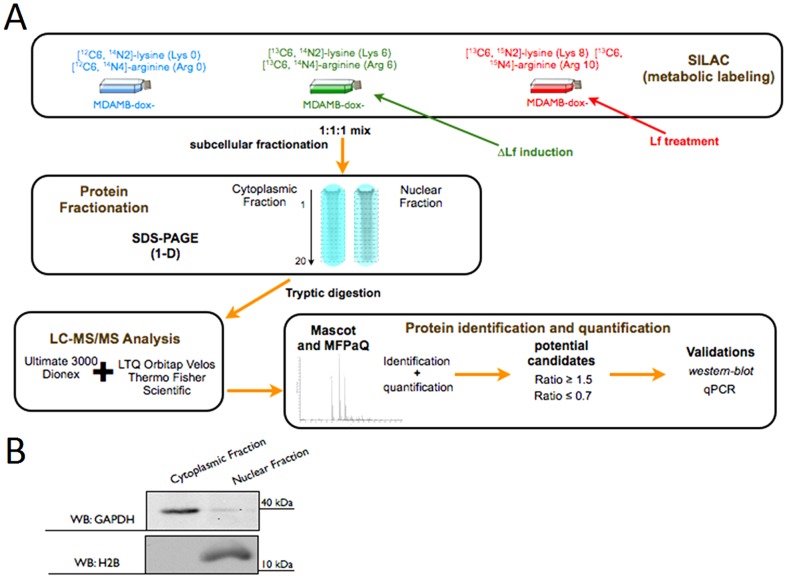
Experimental workflow for triple SILAC. (A) The MDA-MB-231 dox- cells were grown in a defined medium, as described in the experimental section, complemented with essential amino acids Arg and Lys, containing naturally occurring atoms (the light medium) or two of their stable isotope counterparts (the medium and heavy media). The medium culture contained arginine (L-Arg ^13^C_6_-^14^N_4_) and lysine (L-Lys ^13^C_6_-^15^N_2_) and the heavy culture contained arginine (L-Arg ^13^C_6_-^15^N_4_) and lysine (L-Lys ^13^C_6_-^15^N_2_) amino acids. After eight cell divisions to obtain full incorporation of the labeled amino acids into the proteome, cells were then stimulated or not with Lf isoforms. Equal amounts of cells from each condition were combined, creating a single sample that was then subjected to two fractionations. First, subcellular fractionation into cytosolic and nuclear fractions was carried out. In a second fractionation proteins in each subcellular fraction were separated by 1D PAGE. The gel was cut into 20 slices, proteins were digested in the gel slices with trypsin and the resulting peptides extracted from each gel slice were analyzed by reversed-phase nanoscale liquid chromatography (LC) coupled to tandem mass spectrometry (MS/MS). The peptides were electrosprayed into the source of a linear ion trap-orbitrap mass spectrometer (LTQ-orbitrap velos). Bioinformatic analyses were assessed using MFPAQ software that processes the results of the Mascot search engine and performs protein quantification. SILAC light/medium/heavy ratios were assessed by MFPAQ for protein quantification. (B) The subcellular fractionation for the SILAC-screen was assessed using marker proteins of known localization. The markers used were GAPDH for the cytosolic fraction and histone H2B for the nuclear fraction.

To globally assess changes in the proteome of MDA-MB-231 cells stimulated with both Lf isoforms, SILAC coupled to LC-MS/MS for protein identification and quantification was used. SILAC relies on the metabolic incorporation of distinct stable isotope labeled amino acids into the proteome, allowing the discrimination of peptides originating from the differentially treated cell populations by mass spectrometry. We chose a triple SILAC in order to directly compare the differential effects of the re-introduction of hLf or ΔLf versus untreated cancerous cells (Fig. 1A). Cells were harvested 24 h post-stimulation. This time point was chosen since both cell cycle arrest and the beginning of the apoptotic processes are visible. A longer time of treatment may lead to cell mortality and cell viability was therefore controlled using the Trypan blue exclusion method (data not shown).

The complexity of the crude extract of solubilized proteins from MDA-MB-231 cells prevented efficient mass spectrometry (MS) fragmentation analyses and thus the identification of individual proteins. We therefore decided to apply a fractionation process based on the subcellular location of proteins. The three differentially labeled sets of cells were mixed at a 1∶1∶1 ratio and nuclear and cytoplasmic fractions were purified. After protein extraction of each fraction, the homogeneity of the nuclear and cytoplasmic fractions was surveyed using specific markers of nuclear and cytoplasmic compartments (Fig. 1B). Proteins were then separated by SDS-PAGE and digested with trypsin prior to LC-MS/MS analysis. Protein quantification was carried out using MFPAQ [54] and manually validated. An analysis was performed in parallel to measure the label incorporation for each protein quantified in the SILAC analysis. The results from this test-analysis were used to define the accuracy of protein quantification and the protein up-regulation threshold. In good agreement with the expected value of 1, the measured mean ratio of protein mixing remained 1∶1∶1 for proteins from all the three samples indicating that the accuracy of quantitation was not compromised by incomplete labeling. A conservative ratio threshold of 1.5-fold increase (50% higher than the protein mixing error) or 0.7-fold decrease (30% lower than the protein mixing error) in protein abundance above basal level in MDA-MB-231 dox- upon ΔLf or hLf stimulation was considered significant.

Our analysis revealed a total of 5030 identified proteins when low doses of hLf or ΔLf were used (Tables S2-S3). After manual validation taking into account protein identification, the quality of the MS (for quantification) and MS2 spectra (for peptide sequencing) analysis by MFPAQ software, 3 proteins up-regulated in the presence of ΔLf and 8 proteins up-regulated in presence of hLf with a very high confidence were distinguished (Table 1). Moreover, 12 proteins were down-regulated in the presence of ΔLf and 11 were down-regulated in presence of hLf (Table S4).

**Table 1:** Validation of SILAC and mass spectrometry results by Western-blotting and qRT-PCR of differentially expressed proteins in response to low doses of both Lf isoforms.

Accession number	Description	SILAC ratio		Western blot densitometry		Transcript ratio	
		ΔLf	hLf	ΔLf	hLf	ΔLf	hLf
B5MCT8	40S ribosomal protein S9 (RPS9)	2.1	1.9	1.8	1.8	1.0	0.9
P54886	Aldehyde dehydrogenase 18 family, member A1 (ALDH18A1)	2.1	1.6	1.9	1.9	1.0	0.9
Q8IZQ5	Selenoprotein H (SELH)	1.6	1.6	-	-	1.7	1.5
Q92820	Gamma-glutamyl hydrolase (GGH)	1.1	2.2	1.0	2.3	1.0	2.8
Q9UBR2	Cathepsin Z (CTSZ)	1.0	2.5	-	2.1	1.3	2.8
A8K6A7	Mannosidase, alpha, class 2B, member 1 (MAN2B1)	1.0	2.0	-	1.9	1.3	3.3
D6RAQ1	Heparanase (HPSE)	1.0	1.8	1.1	2.0	1.0	2.1
A8K1M2	Calmodulin 1 (CALM1)	1.0	1.6	0.9	1.5	1.0	1.01

The comparative analysis of the proteome profiles of MDA-MB-231 cells treated with a higher dose of Lf isoforms led to the identification of 5309 proteins (Tables S5–S6) with 304 proteins up-regulated for ΔLf (Tables S7–S8), 187 proteins up-regulated for hLf (Tables S9–S10), 217 proteins down-regulated in response to ΔLf (Table S11) and 41 proteins downregulated in response to hLf (Table S12).

### Treatment of MDA-MB-231 cells with a low amount of either hLf or ΔLf

To verify the quantification of proteins in SILAC-based proteomics, the expression of proteins of interest was measured by Western blotting using specific antibodies as described in the experimental section. SILAC and immunoblot-based relative quantifications were in agreement (Table 1, Fig. S1), indicating that the triple SILAC method is a suitable approach to study the effect of Lf isoforms on the MDA-MB-231 proteome. We next investigated whether the protein changes were also visible at the mRNA level (Table 1).

Our first SILAC experiment (Table 1) corresponding to the re-introduction by a low amount of Lf isoforms showed very few proteomic changes. Three proteins were found upregulated either in the presence of hLf or ΔLf: aldehyde dehydrogenase 18 family member A1 (ALDH18A1), ribosomal protein S9 (RPS9) and selenoprotein H (SelH). SelH was upregulated at both the mRNA and protein levels. In contrast, ALDH18A1 and RPS9 were only upregulated at the protein level suggesting the possibility of post-transcriptional events such as enhanced capacity of mRNA translation or increased protein stability. Increased expressions of ALDH18A1 and RPS9 were still visible when a high dose of ΔLf was used (Table 2, Fig. S1).

**Table 2:** Validation of SILAC and mass spectrometry results by Western-blotting and qRT-PCR of differentially expressed proteins in response to high doses of both Lf isoforms.

Accession number	Description	SILAC ratio		Western blot densitometry		Transcript ratio	
		ΔLf	hLf	ΔLf	hLf	ΔLf	hLf
Q53F35	Acidic leucine-rich nuclear phosphoprotein 32B (ANP32B)	9.3	0.4	4.3	0.2	0.9	0.9
Q95218	Zinc finger Ran-binding domain-containing protein 2 (ZNF265)	5.7	0.5	3.1	0.7	4.2	0.8
Q92541	RNA polymerase-associated protein homolog 1 (RTF1)	5.2	0.5	3.6	0.5	0.9	1.0
P13984	General transcription factor IIF subunit 2 (GTF2F2)	4.7	0.4	5.1	0.1	2.5	0.9
P60228	Eukaryotic translation initiation factor 3 subunit E (eIF3E)	3.9	1.2	4.2	1.6	2.2	0.9
P51965	Ubiquitin-conjugating enzyme E2 E1 (UBE2E1)	3.4	0.7	2.9	0.85	2.4	1.0
Q07065	Cytoskeleton-associated protein 4 (CKAP4)	2.6	2.0	2.4	1.9	1.0	2.1
P51665	26S proteasome non-ATPase regulatory subunit 7 (PSMD7)	2.0	1.6	1.9	1.9	0.7	0.9
P54886	Aldehyde dehydrogenase 18 family, member A1 (ALDH18A1)	1.8	1.2	1.9	1.9	1.0	0.9
B5MCT8	40S ribosomal protein S9 (RPS9)	3.8	1.4	1.8	1.8	1.0	0.9
Q9UBR2	Cathepsin Z (CTSZ)	1.4	4	-	2.1	1.2	2.4
Q92820	Gamma-glutamyl hydrolase (GGH)	1.3	3	1.0	2.3	1.0	3.1

SelH is a nucleolar thioredoxin fold-like protein that increases levels of glutathione, glutathione peroxidase activities and antioxidant capacities [59], [60]. ALDH18A1 is a protein implicated in proline and ornithine biosynthesis, and its expression is responsible for cellular ROS downregulation [61]. RPS9 is implicated in ribosomal biogenesis, has a protective role in the apoptotic process and exerts a protective mechanism against oxidative injury [62]. Interestingly, these proteins are all involved in protection against oxidative stress. Modification of the cellular redox environment can be critical for apoptosis induction and production of antioxidant molecules can protect against apoptosis and might correspond to an early defense mechanism of the cancerous mammary gland MDA-MB-231 cells against anti-tumoral agents such as the Lf isoforms.

Seven proteins were found upregulated only in the presence of hLf, four of them were upregulated at both the mRNA and protein levels and possess putative ΔLfRE (Tables 1 and 3). Strangely, some of these proteins seem to exert protumoral effects. Cathepsin Z (CTSZ), a carboxypeptidase degrading heparin sulfate proteoglycans when overexpressed, contributes to tumor metastasis by inducing an epithelial-mesenchymal transition in hepatocellular carcinoma and correlates with an advanced tumor stage [63]. Gamma glutamyl hydrolase (GGH), an acidic enzyme that maintains the homeostasis of folates within the cell, has been shown recently to play a role in the development and progression of invasive breast cancer [64]. Heparanase is a mammalian heparan sulfate degrading enzyme preferentially expressed in highly metastatic human cell lines and in biopsies of human tumors associated with an aggressive malignant phenotype and poor prognosis in cancer patients [65]. Increased expression of both CTSZ and GGH by 3-4-fold were also visible when a high dose of hLf was used (Table 2, Fig. S1). Further work has to be done to clarify whether hLf directly or indirectly upregulates GGH, CTSZ and heparanase expressions.

**Table 3:** ΔLfRE-like sequences found in the promoters of genes regulated by ΔLf and/or hLf.

Promoter	Sequence	Location* (pb)	Accession number/references
S1	G G C A C T - T A/G C		[43]
ΔLfRE *^Skp1^*	G G C A C T G T A C		[13]
ΔLfRE *^DcpS^*	A G C A C T - T G G		[47]
ΔLfRE *^Bax^*	G G C A C T - T A T		[42]
*SelH*	G G C A C T G T G G	−2815	ENSG00000211450
*ALDH18A1*	A G C A C T - T A G	−741	ENSG00000059573
*RPS9*	A G C A C T - T G G	−478	ENSG00000170889
*CTSZ*	G G C A C T - T G G	−3392 R**	ENSG00000101160
*GGH*	A G C A C T T TG G	−3243	ENSG00000137563
*MAN2B1*	A G C A C T T T G G	−1890	ENSG00000104774
*HPSE*	A G C A C T T T G G	−3071	ENSG00000173083
*GTF2F2*	A G C A C T - T G G	−4361	ENSG00000188342
*GTF2F1*	G G C A C T - T G G	−2873	ENSG00000125651
*UBE2E1*	A G C A C T T T G G	−3256 R**, −2787 R**	ENSG00000170142
*CKAP4*	G G C A C T G T G T	−3569	ENSG00000136026
*CKAP4*	A G C A C T - T A T	−2277	ENSG00000136026
*EIF3E*	G A C A C T - T A T	−210	ENSG00000104408
*ANP32B*	A G C A C T - T G G	−3479	ENSG00000136938
*ZNF265*	G G C A C T G T A T	−2578	ENSG00000132485
*RTF1*	AG C A C T T T G G	−4333, −390	ENSG00000137815
*PSMD7*	A G C A C T T T G G	−2789, −2040	ENSG00000103035
*IFIT2*	G G C A C T G T G C	−3995R**	ENSG00000119922
*IFIT2*	A G C A C T - T A G	−3219	ENSG00000119922
*IFIT3*	A G C A C T T T G G	−593	ENSG00000119917
*IFIT1*	A T C A C T - T G G	− −4519 R**	ENSG00000185745
*IFIT1*	A G C A C T G T G G	− −4182 R**	ENSG00000185745
*ISG15*	A G C A C T T T G G	−3991	ENSG00000187608
*IFITM1*	G G C A C T - T G G	−4435R**	ENSG00000185885
*IFITM1*	A G C A C T - T G C	−3279R**	ENSG00000185885

*: Location is relative to the transcription start site.

**R: consensus sequence present on the reverse strand.

The inhibition by hLf of the growth of cancer cells contradicts the notion that hLf may favor malignization and increases synthesis of protumoral proteins. Therefore, we performed an invasiveness assay in order to evaluate whether hLf alters the migration potential of MDA-MB-231 cells. Our preliminary results showed that the penetration of MDA-MB-231 cells into reconstituted basement membrane gel using Matrigel was increased by 2.5-fold in the presence of 50 µg/mL of hLf and by a 3-fold with 500 µg/mL of hLf (data not shown) and even if only 0.2–0.3% of the cells are concerned by this process, this could be quite important in terms of invasiveness and formation of metastases. We cannot explain our result concerning the invasion assay since a large number of *in vitro* and *in vivo* studies agree on the *anti*-metastatic activities of human and bovine Lf. Since 1994 and the work of Bezault et al. [23], it is known that Lf may exert anti-metastatic activity. Thereafter many studies, including animal models of chemical carcinogenesis, have confirmed this property. However, Oh et al. [66] have shown that Lf is nevertheless capable of increasing the expression of metalloproteinases such as MMP1. Their study showed that Lf is able to promote cell motility indirectly, which is in contradiction with its potential to inhibit metastasis formation. Recently, Ha et al. [45] have shown that hLf treatment (100 µg/mL) of several mammary cancer cell lines including MDA-MB-231 cells has the capacity to increase migration and invasion of the treated cells, mediated *via* the transcriptional activation of the *endothelin-1* gene. MMP1 and endothelin-1, a secreted pro-invasive polypeptide were not detected in our SILAC assays. Further work has to be done to see whether an overexpression of heparanase and cathepsin Z in response to hLf might be responsible for the degradation of extracellular matrix proteoglycans and increased invasiveness.

On the other hand, proteins of the S100 family were down-regulated (Table S4; Fig. S1). S100 proteins are a group of small acidic calcium-binding proteins interacting with cytoskeletal proteins, transcription factors, and nucleic acids to regulate cell cycle progression, differentiation, apoptosis, cell migration, inflammation and calcium homeostasis. Altered expression of many S100 proteins such as S100-A6, S100-A9 and S100-A7 has been reported associated with tumor progression and metastasis in several types of cancer including breast cancer [67]. Thus, the down-regulated expression of these pro-invasive proteins confirms that both Lf isoforms exert anti-tumoral activity. Lf and ΔLf, when feebly expressed, may control cell homeostasis by finely regulating the balance between pro- and anti-tumoral events. This duality illustrates the complexity of the understanding of the overall Lf functions. Lf itself was found downregulated whatever Lf or ΔLf doses used (Tables S4, S11, S12). This may be due to a negative feedback mechanism in order to reduce cell sensitivity to Lfs. This downregulation may be achieved by enhanced post-transcriptional and/or post-translational degradation since Lf and ΔLf transcription levels were not altered (Fig. S2).

### Treatment of MDA-MB-231 cells with high amounts of either hLf or ΔLf

The upregulated proteins identified in the second SILAC experiment concerning cells treated with high amounts of hLf or ΔLf are summarized in Tables S5–S6. Among the upregulated proteins, 134 were common to both hLf and ΔLf treatments whereas 53 proteins were only identified in hLf treated cell extracts and 170 in ΔLf expressing cell extracts. As already observed for the first SILAC experiment, SILAC and immunoblot-based relative quantifications were in agreement for the second assay with higher doses of Lf isoforms (Table 2, Fig. S1). We also carried out qRT-PCR experiments and *in silico* analyses on the corresponding promoters of the most highly upregulated proteins (Tables 2 and 3). Among the most highly upregulated proteins in ΔLf expressing MDA-MB-231 cytoplasmic extract, three members of the interferon-induced protein with tetratricopeptide repeats (IFIT) family were found (IFIT2, 56-fold; IFIT3, 15.3-fold; IFIT1, 14-fold; Table S8). Their gene promoters possess ΔLfRE-like sequences the functionality of which has to be confirmed. IFIT family members, known to respond to infections, have been recently described as inhibitors of cell migration and proliferation [68]. Elevated IFIT1 protein expression in breast cancer is associated with improved local relapse-free survival [69]. Knockdown of IFIT2 expression in oral squamous cell carcinoma leads to tumor progression, particularly during metastasis due to increased migration rates [70], [71]. Moreover, IFIT2 expression promotes cellular apoptosis [72]. IFIT1 and IFIT2 antitumoral functions are notably due to their interaction with a variety of cytoskeleton molecules and to their interaction with eIF3 leading to translation inhibition [73]. Antiproliferative activity has also been ascribed to IFIT3 expression, which leads to upregulation of P27 and P21 and cell accumulation at the G_1_ phase [74]. Interestingly IFIT1 function and stability are modulated by conjugation to the ubiquitin-like protein ISG15 [75], which is itself upregulated in ΔLf expressing cells by 12-fold (Table S8). There is strong evidence that in some cancers immunosurveillance plays an integral role in tumor initiation and growth. Therefore by upregulating IFIT members, ΔLf may contribute directly or indirectly to an increase in the immune response and host cell protection against tumorigenesis. Further work will be needed to correlate ΔLf and IFITs expressions with immunoprotection against breast cancer.

**Figure 2:**
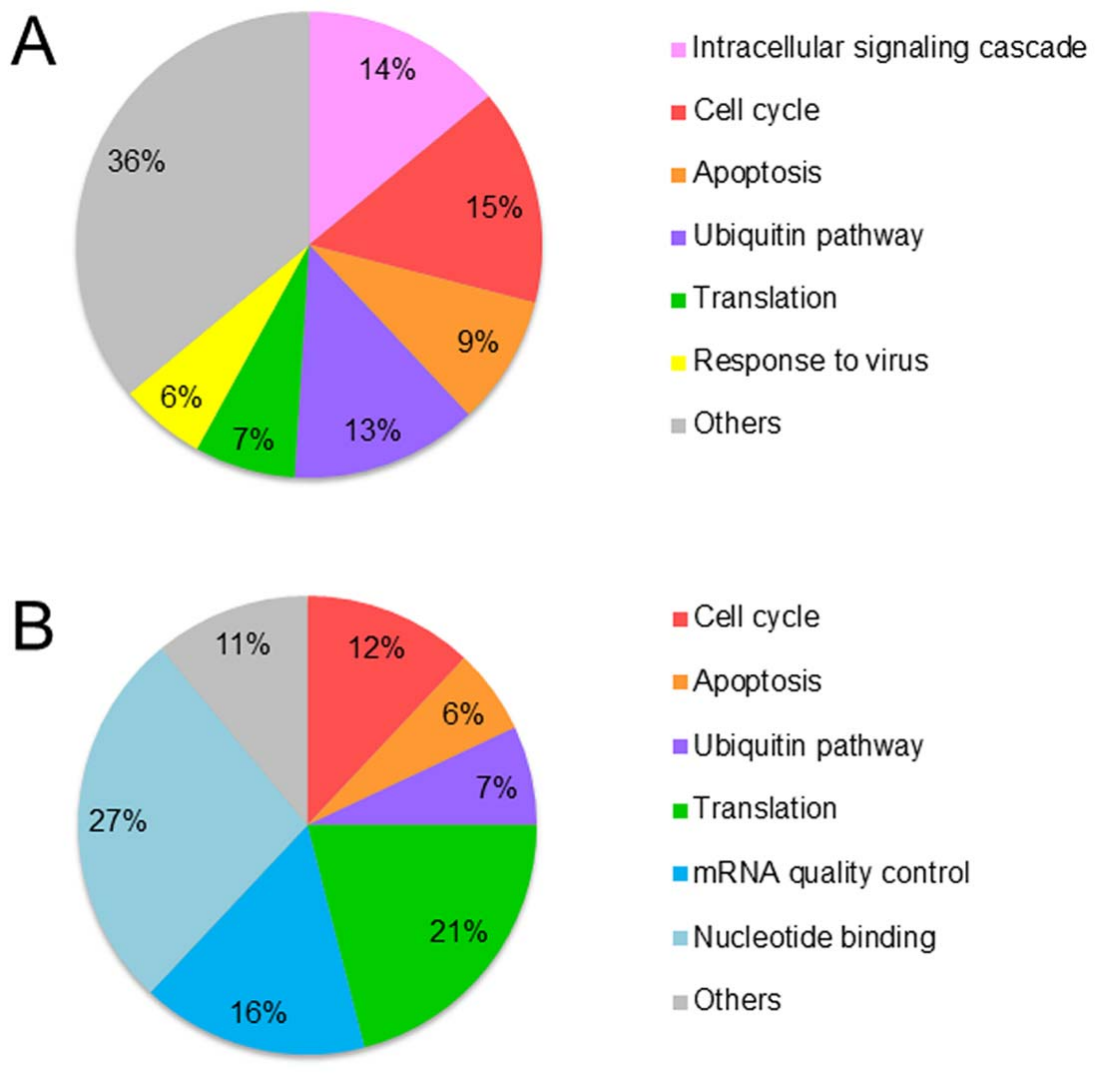
Overall evaluation of overexpressed protein identities. Pie chart representations of the classification by biological processes of the proteins (as identified by nano-LC Orbitrap-MS/MS) found in response to hLf treatment (A) and ΔLf induction (B). Proteins were classified using the DAVID classification system (http://david.abcc.ncifcrf.gov/). All the categories are statistically significant with P- value, 0.01.

Since this second SILAC experiment showed very large proteomic changes we carried out analyses using the Database for Annotation, Visualization and Integrated Discovery (DAVID). The Uniprot accession numbers were uploaded to the DAVID tools and upregulated nuclear and cytosolic proteins were classified by molecular functions and biological process. The classification of the proteins upregulated in the presence of hLf (Fig. 2A) showed that approximately 60% were involved in the regulation of cellular processes among which 40% of this protein pool was involved in the maintenance of cellular homeostasis with proteins involved in cell signaling, the cell cycle and apoptosis. These data are in accordance with what is known of Lf function [22]. Interestingly, some of the identified proteins are involved in protein turnover: synthesis and degradation. The classification of the proteins upregulated in the presence of ΔLf (Fig. 2B) showed that approximately 90% were involved in the regulation of cellular processes. Twenty percent of them are involved in the maintenance of cell homeostasis notably cell cycle regulation and apoptosis. Again, these data are in accordance with our knowledge of ΔLf function [11]. Interestingly, 65% of the proteins identified are involved in the control of protein quantity: mRNA quality control, transcription, nucleotide binding and translation. *Skp1*, *Bax* and *DcpS*, the target genes of ΔLf transcriptional activity we characterized previously, belong to these two groups of proteins.

**Figure 3:**
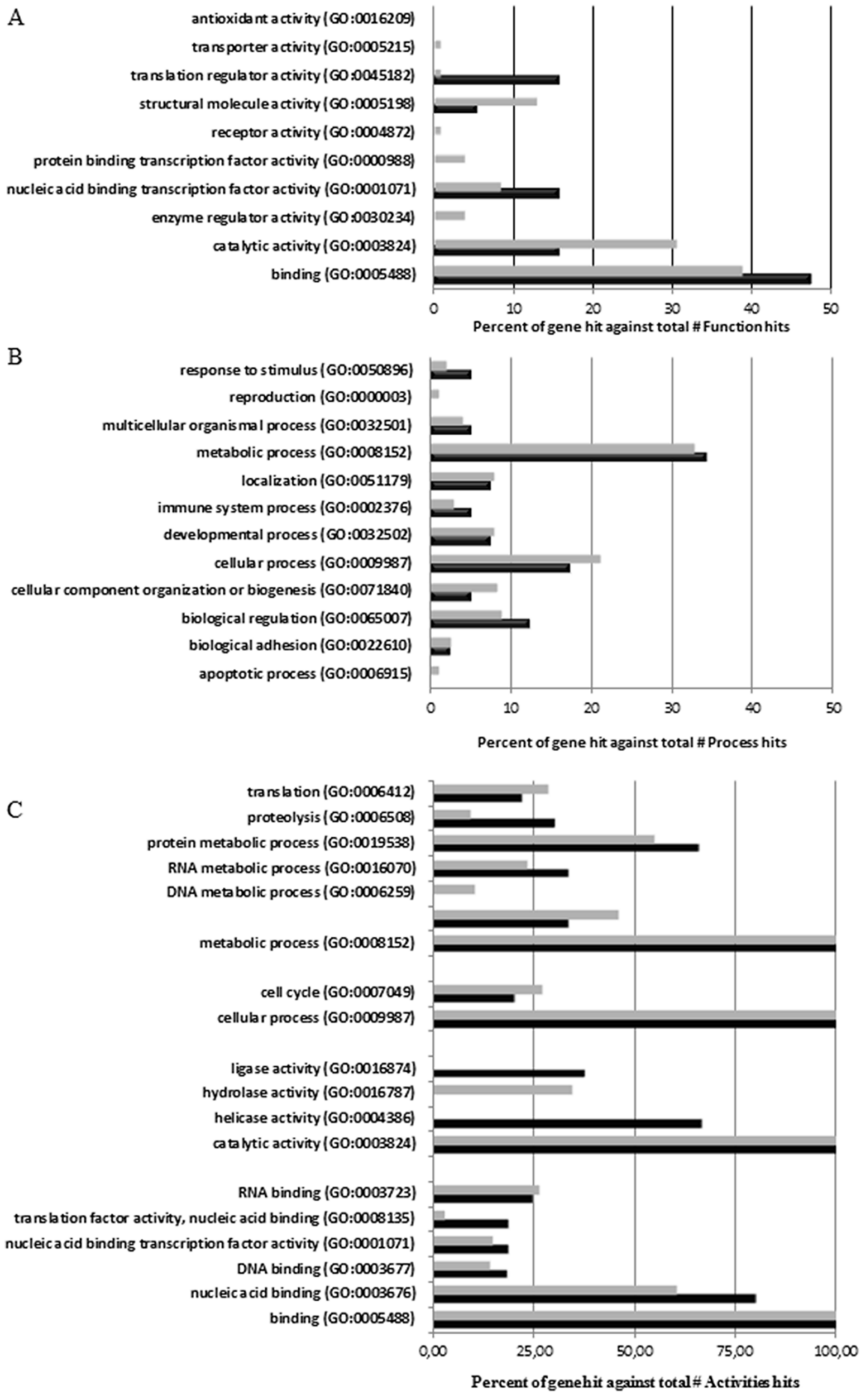
Overall evaluation of downexpressed protein identities. Histogram representations of the distribution of the downregulated proteins in response to hLf treatment and ΔLf induction according to their molecular functions (A) and biological processes (B). Gene Ontology categorizations were based on information provided by the online resource PANTHER 9.0 classification system (http://www.pantherdb.org/). Panel (C) shows lower level classifications of biological processes and molecular functions for Lf isoforms regulated responsive genes. Gene Ontology lower level categorizations were expressed as percentages: 100% corresponds to metabolic process, cellular process, catalytic activity or binding categorization, respectively. All the categories are statistically significant with P- value, 0.01.

The downregulated proteins are summarized in Tables S11 for ΔLf and S12 for hLf. Among them, 18 were common to both hLf and ΔLf treatments whereas 23 proteins were only identified in hLf treated cell extracts and 199 in ΔLf expressing cell extracts. To better characterize the downregulated proteins, we classified them into functional categories according to the PANTHER system. These proteins are implicated in a broad range of molecular functions (Fig. 3A) and biological activities (Fig. 3B). ΔLf and Lf downregulated genes are mainly involved in molecular functions such as catalytic activity and binding. We next expanded our study on lower level terms that allow us to identify specific functional categories (Fig. 3C). Binding for both isoforms was mainly nucleic acid binding (80% for Lf and 60% for ΔLf) and notably mRNA binding (30% for Lf and 45% for ΔLf). The catalytic activity category corresponded for Lf to genes mainly involved in helicase (70%) and ligase (30%) activities and for ΔLf to genes with hydrolase activities (35%). The gene ontology tree for biological process showed that the highest percentages of ΔLf and Lf downregulated genes were involved in primary metabolic processes among which nucleobase-containing compound metabolic process and more specifically RNA metabolic process and protein metabolic process and notably proteolysis and translation categories are the most represented. Our data suggest that the cancer-inhibitory effect of Lf isoforms may also rely on the down-regulation of genes involved in nucleic acid binding, mRNA processing, protein turn-over, translation and mitosis.

### Both ΔLf and transfected hLf act as transcription factors and target the *SelH* promoter

**Figure 4:**
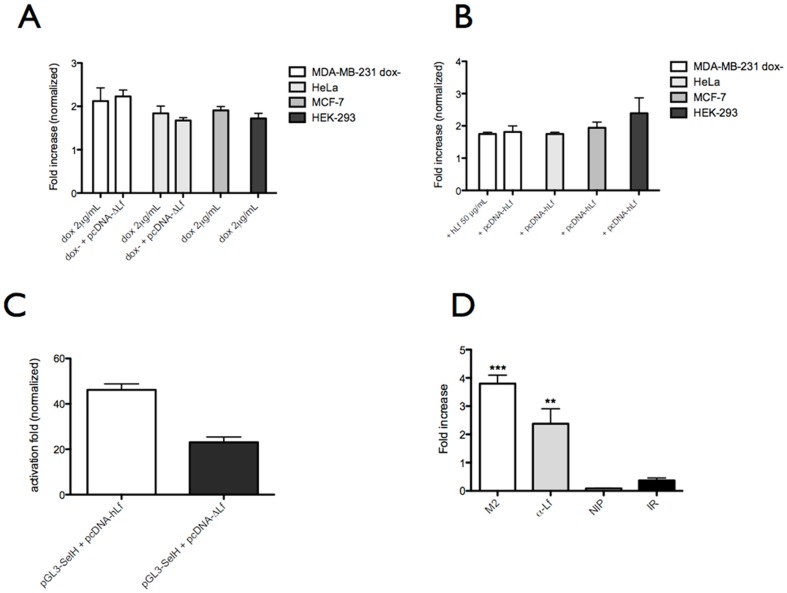
Both ΔLf and hLf act as transcription factors and target the *SelH* promoter. Panels A and B. *SelH* mRNA overexpression is not cell specific. MDA-MB 231, HeLa, MCF7 and HEK 293 cells were induced with doxycycline (2 µg/mL) or transfected with pcDNA-ΔLf (A). MDA-MB 231, HeLa, MCF7 and HEK 293 cells were transfected with pcDNA-hLf whereas only MDA-MB 231 cells were treated with exogenous hLf (50 µg/mL) (B). The expression pattern of *SelH* transcripts in MDA-MB-231, HeLa, MCF-7 and HEK-293 cells after treatment was followed by qRT-PCR. Data are normalized to HPRT and are expressed as the fold increase (2^−ΔΔCt^) under ΔLf (A) or hLf (B) treatment (n = 3). Panel C. HEK 293 cells were cotransfected with pGL3-SelH-Luc construct (50 ng/well) and pcDNA-hLf expression vector (200 ng/well) encoding intracellular hLf or pcDNA-ΔLf expression vector (200 ng/well) encoding ΔLf. 24 h after transfection, cells were lysed and samples were assayed for protein content and luciferase activity. The relative luciferase activity reported is expressed as the fold increase of the ratio of the pGL3 reporter activity to protein content. Values represent the mean ±SD of triplicates from three independent measurements. Panel D. ΔLf and hLf are recruited *in vivo* on the *SelH* promoter. HEK 293 cells were transfected with the pcDNA-hLf or the pCMV-3XFLAG-ΔLf. 24 h post transfection, ChIP assays were performed, using an anti-FLAG (M2), an anti-hLf (α-Lf) and anti-rabbit IgG as nonspecific antibody control (IR). As a further control, the assay was performed without binding of an antibody to the protein G plus Sepharose (NIP). The isolated genomic DNA was analyzed by real time PCR using primers that link the ΔLfRE binding site on the *SelH* promoter. The results were normalized with the levels of ΔLfRE present in the samples (input). Data are expressed as fold enrichment related to null-transfected cells, and are the mean ±SD of triplicates from three independent assays. *p<0.05; **p<0.01; ***p<0.001.

SelH was upregulated at the mRNA and protein levels in the presence of either ΔLf or secreted hLf (Table 1). In order to study the cell specificity of *SelH* overexpression in the presence of both Lf isoforms, the level of *SelH* mRNA expression was measured by qRT-PCR in MDA-MB-231, HeLa, MCF-7 and HEK-293 cell lines in which ΔLf was either transiently or stably expressed and hLf added in the culture medium or transiently transfected. The hLf-expressing vector construct was used with cells for which hLf uptake was feeble or not described. As shown in Fig. 4A and 4B, a nearly 2-fold increase was observed in all cellular models for *SelH* mRNA confirming that *SelH* overexpression is not cell specific.

We next investigated the putative existence of a ΔLfRE in the promoter of *SelH* in order to find out whether it may be a potential target of hLf/ΔLf transcriptional activity. Our *in silico* study pointed out a ΔLfRE sequence identical to that found in the *Skp1* promoter (Table 3). The functionality of the ΔLfRE of the *SelH* promoter was confirmed both using a luciferase reporter gene (Fig. 4C) and ChIP (Fig 4D) assays. Fig. 4C shows that plasmids expressing intracellular ΔLf and hLf when transfected into HEK293 cells, induce a marked increase in luciferase activity after binding to the *SelH* enhancer/promoter region. Gene transactivation by ΔLf led to a 20-fold increase whereas a 40-fold increase was seen in the presence of intracellular hLf. The higher transactivation response with hLf might be due to the presence of two NLS instead of one in ΔLf which may lead to higher or/and faster delivery of hLf into the nucleus. Figure 4D shows that cytoplasmic hLf and ΔLf bind to the human *SelH* promoter *in vivo*. To this end, a 3XFLAG-N-terminus-tagged ΔLf was used to obtain the most reliable results [15]. After immunoprecipitation by M2 (anti-FLAG epitope) or anti-hLf antibodies, PCR amplification with the *SelH*-specific primers revealed an enrichment of the *SelH* promoter region by 4-fold with ΔLf and by 2.5-fold with hLf (lanes 1–2, Fig. 4D). Control experiments involving non-specific antibodies showed only a slight amplification of the PCR product (lane 4, Fig. 4D) confirming the specificity of the results, which was reinforced by the loading control, corresponding to the immunoprecipitation of chromatin with pure protein G Plus Sepharose (lane 3, Fig. 4D). In conclusion, ΔLf and endogeneous hLf act as transcription factors whereas exogeneous hLf does not in HEK 293 cells (Fig 4B). Although surface nucleolin is ubiquitously expressed at the surface of dividing cells [76] we performed another ChIP assay with MDA-MB-231 cells. Despite this change we were unable to demonstrate that exogeneous hLf either at 50 or 500 µg/mL binds the *SelH* promoter (data not shown). Legrand *et al*. previously demonstrated that Lf colocalizes with surface nucleolin on MDA-MB-231 cells and together they become internalized through vesicles of the recycling–degradation pathway by an active process and that only a small proportion of Lf translocates into the nucleus of cells [17]. This might explain why it was difficult to obtain a clear answer when performing ChIP assays with exogeneous Lf but we cannot exclude the possibility that induction of overexpression of *SelH* by exogeneous hLf might be an indirect process involving a receptor-mediated signaling pathway. Thus, trans-activation of the *matrix metalloproteinase 1 (MMP1*) gene by hLf is effected through stress-activated MAPK signaling pathways [66].

### *In vivo* recruitment of ΔLf to new target genes, *GTF2F2* and *UBE2E1*

We performed *in silico* analyses of the promoters of some of the genes corresponding to the proteins up-regulated in the presence of ΔLf and among those which were also up-regulated at the mRNA level we selected two in order to investigate whether they were new ΔLf transcriptional targets (Table 3). UBE2E1 (ubiquitin-conjugating enzyme E2E1) is a member of the ubiquitin-conjugating enzyme family, which catalyzes the final attachment of ubiquitin to a substrate protein, often in concert with ubiquitin-ligases E3. The involvement of E2 enzymes in ubiquitin modification pathways reflects their crucial roles in processes such as protein turnover, function, and localization, thereby controlling cell homeostasis [77]. GTF2F2 (RAP30), an ATP-dependent DNA-helicase, belongs to the general transcription factor IIF (TFIIF) and exists as a heterodimer with GTF2F1 (RAP74). The complex has been shown to bind RNA polymerase II, helps to recruit it to the initiation complex and controls the activity of RNA polymerase II in both the initiation and elongation stages of transcription [78].

**Figure 5:**
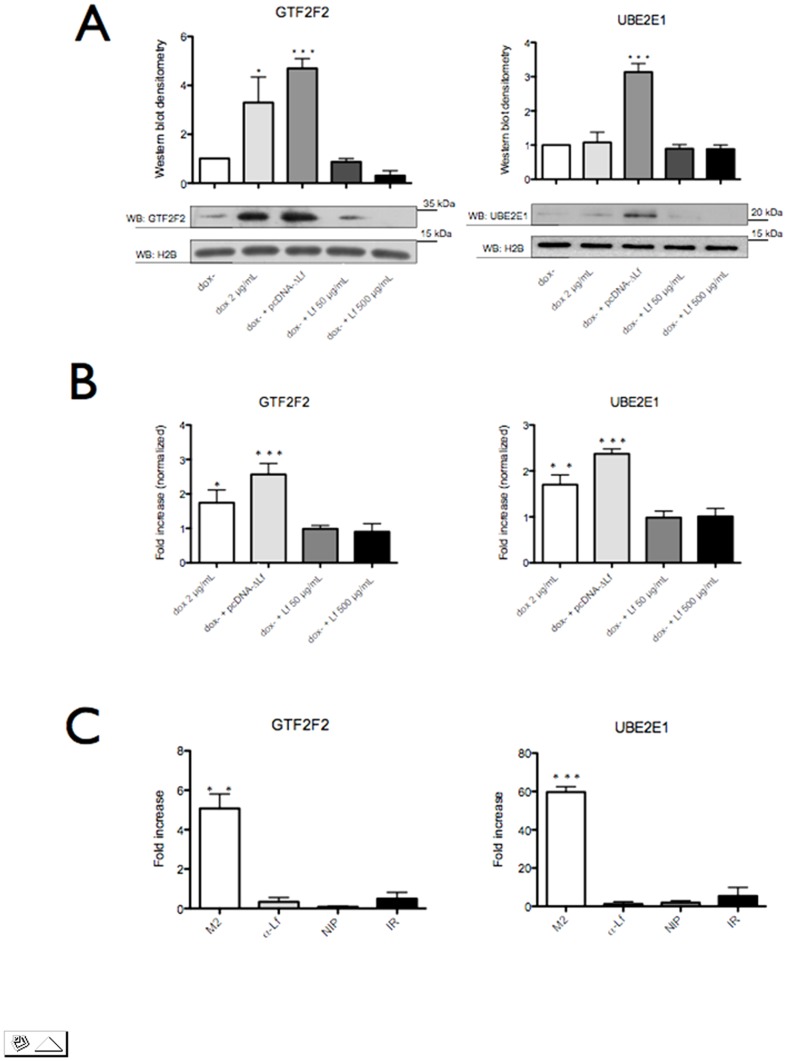
*In vivo* recruitment of ΔLf on new target genes, *GTF2F2* and *UBE2E1*. Panel A. Differential protein expression was confirmed using Western blotting. MDA-MB-231 dox- cells were lysed 24 h after treatment and samples (20 µg of protein) were subjected to SDS-PAGE and immunoblotted with antibodies specific to GTF2F2 and UBE2E1. Histone H2B served as loading control. Panel B. *GTF2F2* and *UBE2E1* mRNA are upregulated in ΔLf-expressing MDA-MB-231 dox- cells. Cells were either untreated or induced with doxycycline (2 µg/mL) or transfected with pcDNA-ΔLf or treated with exogenous hLf (50 or 500 µg/mL). mRNA content was determined by qRT-PCR. Panel C. HEK 293 cells were transfected with the pCMV-3XFLAG-ΔLf or pcDNA-hLf. 24 h post transfection, a ChIP assay was performed. ChIP product was then amplified by real time PCR using specific primer pairs targeting the ΔLfRE containing fragment of the each targeted promoter. ChIP assays were performed using an anti-FLAG (M2), an anti-hLf (α-Lf) and anti-rabbit IgG as nonspecific antibody control (IR). As a further control, the assay was performed without binding of an antibody to the protein G plus Sepharose (NIP). The isolated genomic DNA was analysed by real time PCR using primers specific for ΔLfRE putative binding site on *GTF2F2* and *UBE2E1* promoters. The results were normalized with the levels of ΔLfRE present in the samples (input). Data are expressed as fold enrichment related to null-transfected cells, and are the mean ±SD of triplicates from three independent assays. *p<0.05; **p<0.01; ***p<0.001.

Fig. 5A shows the variations of the expression of GTF2F2 and UBE2E1 when Lf isoforms were re-introduced into MDA-MB-231 cells. GTF2F2 was up-regulated by nearly 3-fold when ΔLf was induced under doxycycline stimulation and by 5-fold when the ΔLf expression vector was transfected. The production of ΔLf also leads to an increased expression of UBE2E1 by 3-fold in the transfected cells. Fig. 5B confirms that overexpression was also visible at the mRNA level with an average 2-2.5 fold increase. Exogeneous hLf treatment whatever concentration used had no effect on these two genes.

The *in silico* study highlighted the presence of putative response elements in the *GTF2F2* and *UBE2E1* promoters (Table 3). We next investigated whether ΔLf interacts *in vivo* with their ΔLfREs and performed ChIP assays. Figure 5C shows a six-fold higher level of amplification product for *GTF2F2* promoter-ΔLf immunoprecipitate in ΔLf-expressing cells as compared to ΔLf-non expressing cells and a sixty-fold higher level of amplification product for the *UBE2E1* promoter. A weak signal was detected in control conditions (NIP and IR) and corresponded to the background inherent to the method. This data demonstrate specific *in vivo* binding of ΔLf to *GTF2F2* and *UBE2E1*, which are therefore transcriptional targets of ΔLf.

## Conclusions

In summary, we present a large scale proteomic study of the response to the two main isoforms of Lf, secreted hLf and nucleocytoplasmic ΔLf. The survey of the MDA-MB-231 proteome in which hLf isoforms have been reintroduced was greatly facilitated by the use of the SILAC strategy. By using a rational combination of quantitative proteomic profiling, antibody-based validation techniques and real time PCR assays, we identified a number of novel potential target proteins with many important cellular functions. Their global analysis may provide insight into the roles of hLf and ΔLf in cell homeostasis. Although these are not *in vivo* studies, the generated data could be used to select important proteins and follow their occurrence and activity in animal models of chemically induced carcinogenesis or transplanted tumors.

## Supporting Information

Figure S1 **SILAC proteins were validated by Western blotting**. 24 h after treatment, MDA-MB-231 cells were lysed. Proteins were extracted and 30 µg of protein were loaded on 10% SDS-PAGE. Western blot detection was performed as described in the experimental section. H2B was used as internal control.
(TIF)

Figure S2 **Expression levels of Lf and ΔLf mRNAs in MDA-MB-231 cells treated with Lf isoforms.** Duplex TaqMan qRT-PCR was performed as described in the experimental section. Grey bars, Lf transcript; black bars, ΔLf transcript. Values are normalized to HPRT gene expression. Data are means ±SD of triplicates from three independent assays. **p<0.01.
(TIF)

Table S1 **DNA primers (F: forward; R: reverse) and conditions used to amplify mRNA and promoter fragments.**
(DOC)

Table S2 **List of nuclear proteins identified when low doses of Lf isoforms are used.**
(XLSX)

Table S3 **List of cytosolic proteins identified when low doses of Lf isoforms are used.**
(XLSX)

Table S4 **List of downregulated proteins identified when low doses of ΔLf and hLf are used.**
(XLSX)

Table S5 **List of nuclear proteins identified when high doses of Lf isoforms are used.**
(XLSX)

Table S6 **List of cytosolic proteins identified when high doses of Lf isoforms are used.**
(XLSX)

Table S7 **List of upregulated nuclear proteins identified when high doses of ΔLf are used.**
(XLSX)

Table S8 **List of upregulated cytosolic proteins identified when high doses of ΔLf are used.**
(XLSX)

Table S9 **List of upregulated nuclear proteins identified when high doses of hLf are used.**
(XLSX)

Table S10 **List of upregulated cytosolic proteins identified when high doses of hLf are used.**
(XLSX)

Table S11 **List of downregulated proteins identified when high doses of ΔLf are used.**
(XLSX)

Table S12 **List of downregulated proteins identified when high doses of hLf are used.**
(XLSX)
