# Supplementary figures and images for: SILAC-Based Proteomic Profiling of the Human MDA-MB-231 Metastatic Breast Cancer Cell Line in Response to the Two Antitumoral Lactoferrin Isoforms: The Secreted Lactoferrin and the Intracellular Delta-Lactoferrin
Source: PLoS One. 2014 Aug 12;9(8):e104563. doi: 10.1371/journal.pone.0104563 (PMC4130549; doi:10.1371/journal.pone.0104563)

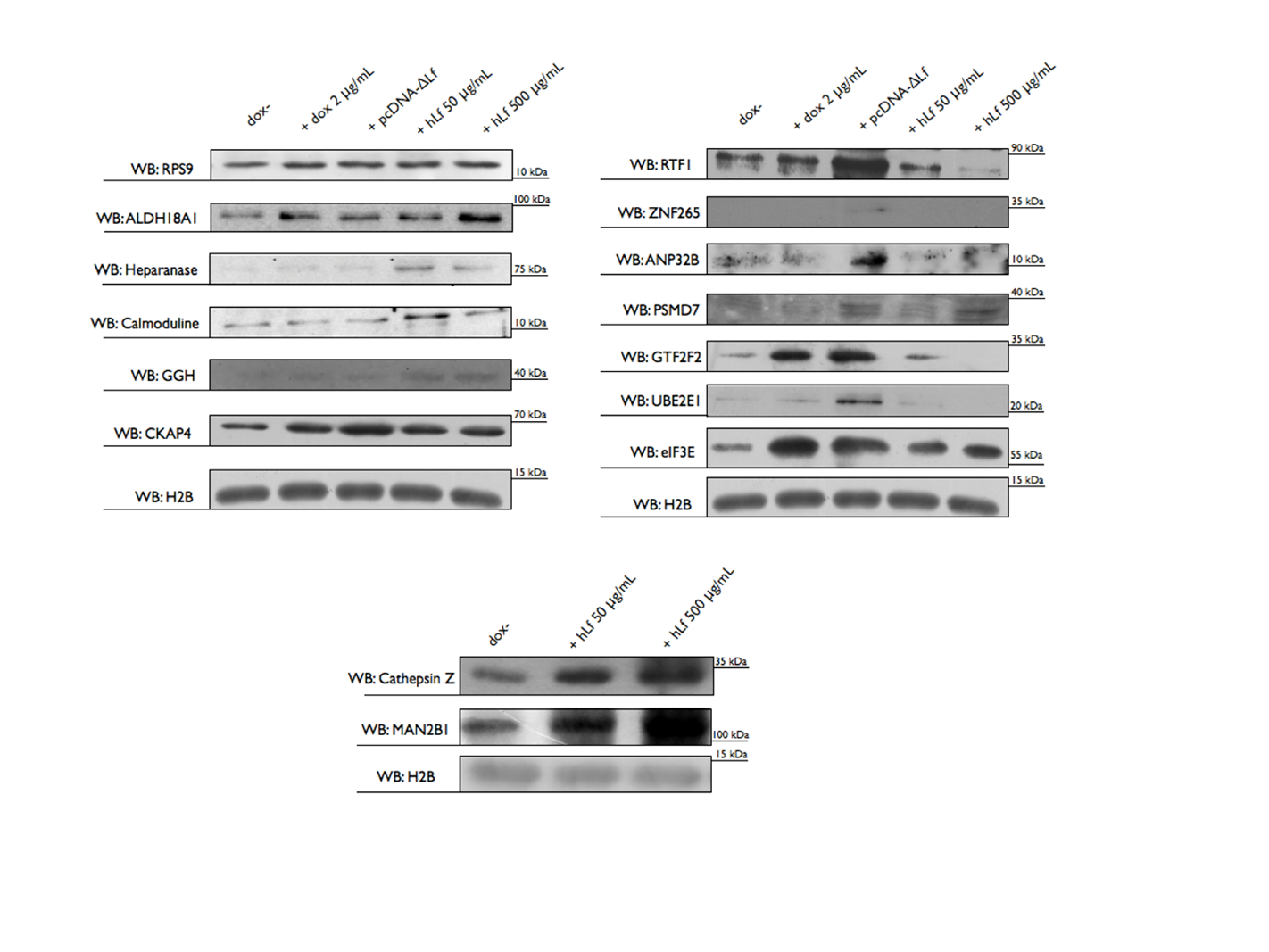

Supplement: Figure S1 — SILAC proteins were validated by Western blotting. 24 h after treatment, MDA-MB-231 cells were lysed. Proteins were extracted and 30 µg of protein were loaded on 10% SDS-PAGE. Western blot detection was performed as described in the experimental section. H2B was used as internal control. (TIF) [file pone.0104563.s001.tif]

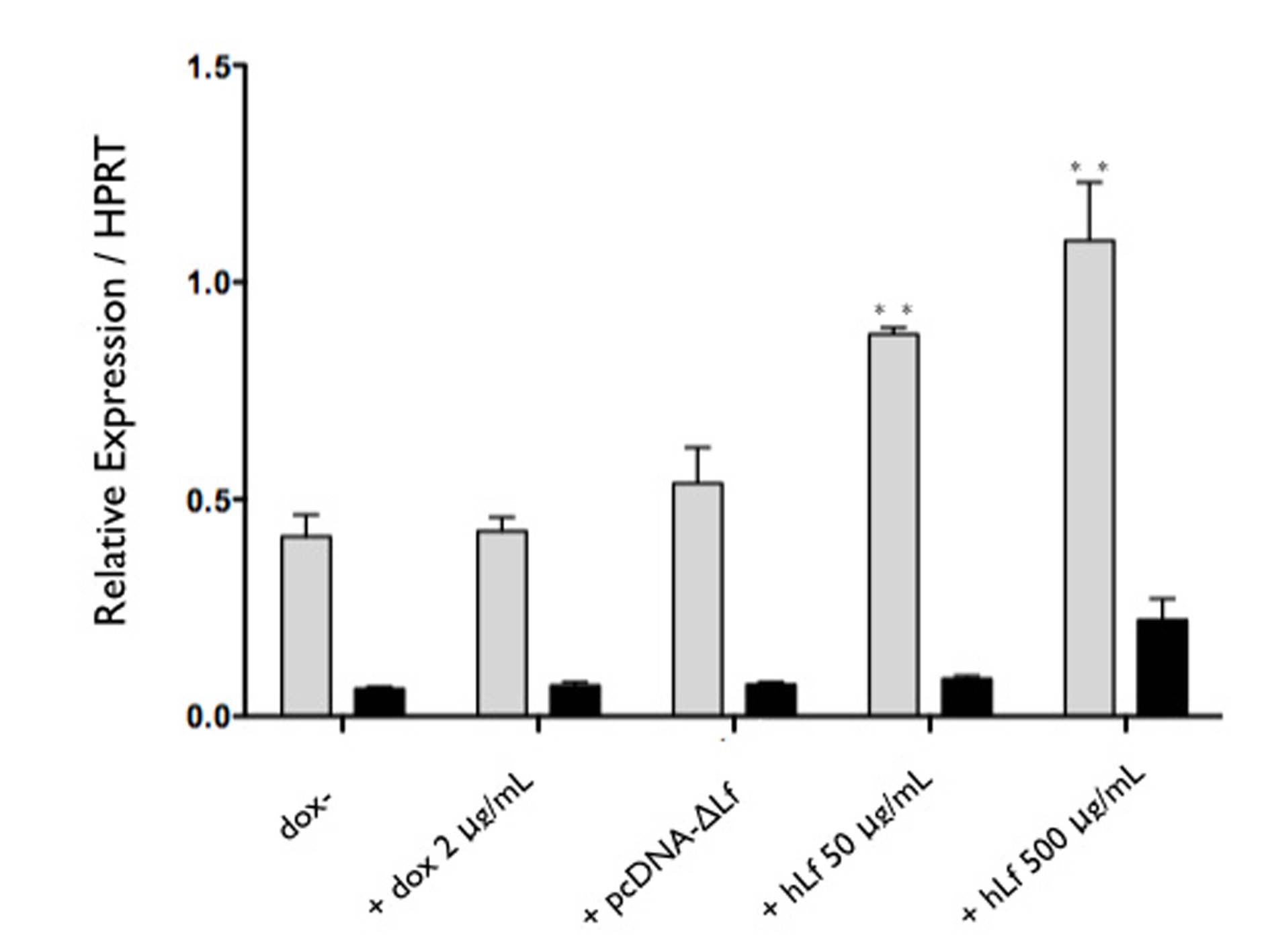

Supplement: Figure S2 — Expression levels of Lf and ΔLf mRNAs in MDA-MB-231 cells treated with Lf isoforms. Duplex TaqMan qRT-PCR was performed as described in the experimental section. Grey bars, Lf transcript; black bars, ΔLf transcript. Values are normalized to HPRT gene expression. Data are means ±SD of triplicates from three independent assays. **p<0.01. (TIF) [file pone.0104563.s002.tif]
