# Supplementary material for: SILAC-Based Proteomic Profiling of the Human MDA-MB-231 Metastatic Breast Cancer Cell Line in Response to the Two Antitumoral Lactoferrin Isoforms: The Secreted Lactoferrin and the Intracellular Delta-Lactoferrin
Source: PLoS One. 2014 Aug 12;9(8):e104563. doi: 10.1371/journal.pone.0104563 (PMC4130549; doi:10.1371/journal.pone.0104563)
Supplement: Table S1 — DNA primers (F: forward; R: reverse) and conditions used to amplify mRNA and promoter fragments. (DOC) [file pone.0104563.s003.doc]

**Table S1.** DNA primers (F: forward; R: reverse) and conditions used to amplify mRNA and promoter fragments.

| **Method** | **Target DNA** | **Oligonucleotides (5’ 3’)** | **Tm (°C)** | **Amplicon size (pb)** |
| --- | --- | --- | --- | --- |
| **qRT-PCR** | RPS9 | F: TGGTTTGCTTAGGCGCAGA R: TACTCGCCGATCAGCTTCAG | 60 | 141 |
|  | SELH | F :GCTTCCAGTAAAGGTGAACCCG R: CCCAAATCTCCCTACGACAGG | 55 | 101 |
|  | ALDH18A1 | F: TGACCTGCAGGGGGTTATTA R: CTTTCACCTTGGCTTCCATGC | 51 | 236 |
|  | Cathepsin Z | F: TGTCCGGAATTCATGGGGTG R: TGGCCTTAAACGATGGGGTC | 60 | 144 |
|  | GGH | F: ACTGCATTTCCCATAGGCCC R: CCTTTCAAAAGCTCTGCGGG | 60 | 116 |
|  | MAN2B1 | F: CTGGTAAATGCGCAGGCAAAA R: TCCGCGTAAGGGAAGAAGTC | 60 | 131 |
|  | Heparanase | F: TGGACCTGGACTTCTTCACC R: TTGATTCCTTCTTGGGATCG | 60 | 118 |
|  | Calmodulin 1 | F: TGAGGTCTCGAGTTCCCTCGGC R: TTGTTGTGATGGTGCCATCGCC | 60 | 331 |
|  | CKAP4 | F: GACGGAGCTCACCAAATCCA R: CTTCCAGGGAGGCAACCTTT | 60 | 110 |
|  | RTF1 | F: GGAACCGGGAGTGGAACATT R: GCTTGAACAGCTGGGTCTCT | 60 | 145 |
|  | ZNF265 | F: CGAAAGCCAGGTGATTGGTG R: GATGATGACCTGTGGCGTCT | 60 | 99 |
|  | ANP32B | F: TCCTGTGACATTCCGCCTTC R: GTAGCCACAGACGGTTGACT | 60 | 163 |
|  | PSMD7 | F: ATCAAAGACACGACGGTGGG R: ACATCTGGCAGCAGGTTGAA | 60 | 185 |
|  | GTF2F2 | F: TGCGGATTGCCAAGACTCAA R: AGGAGCACTGACTGAAGCTG | 60 | 107 |
|  | UBE2E1 | F: GAGAGACACGAGTGGCCAGG R: AAAGTGATGTCCGCCAGCTC | 61 | 296 |
|  | HPRT | F: GATGACCAGCCCAAAGGAGA R: GTGATGTCAGCTGATCAAGACT | 60 | 101 |
|  | EIF3E | F: TTTGGCAAGATGGCGGAGTA R: AGCGGAAAGACTAGATGCCG | 60 | 74 |
| **Plasmid construction** | pGL3-*SelH*-Luc | F: GGAGGAACAACAGTGCTTCAA R: TCATCCTGGTGTTCCCTCTCC | 60 | 167 |
| **ChIP** | *SelH* promoter | F:AGGAGGAACAACAGTGCTTCAA R: TCATCCTGGTGTTCCCTCTCC | 60 | 167 |
|  | *GTF2F2* promoter | F: GCGCCTGGCCTATAGAGTTT R: CTGATTTCAAGCTGCCAAGA | 60 | 94 |
|  | *UBE2E1* promoter | F: ATCTCCTGACCTCGTGTTCC R: TTATGTTGGCCGGTATGTTG | 60 | 96 |
